# Impact of tumor multiplicity on the prognosis of patients with primary renal cell carcinoma: a SEER database analysis

**DOI:** 10.1007/s10238-024-01433-w

**Published:** 2024-08-17

**Authors:** Tianyue Yang, Hongfeng Zheng, Shaojun Chen, Min Gong, Yifan Liu, Wang Zhou, Jianqing Ye, Xiuwu Pan, Xingang Cui

**Affiliations:** 1 Department of Urology, Xinhua Hospital, School of Medicine, Shanghai Jiao Tong University, 1665 Kongjiang Road, Yangpu District, Shanghai, 200092 China; 2 Department of Urology, Seventh People’s Hospital of Shanghai University of Traditional Chinese Medicine, Shanghai, 200137 China

**Keywords:** Renal cell carcinoma, Nomograms, Risk factors, SEER program, Survival analysis

## Abstract

To compare clinical characteristics and survival outcomes of patients with multiple renal cell carcinoma versus single renal cell carcinoma. Develop a prognostic model for predicting prognosis in patients with multiple tumors and analyze prognostic factors. Patients with primary multiple renal cell carcinoma were selected from the Surveillance, Epidemiology, and End Results database (2004–2015). They were divided into single-tumor and multiple-tumor groups. Survival analysis was conducted using the Kaplan–Meier method and log-rank test. A Cox regression model was used to identify potential prognostic factors. A total of 19,489 renal cell carcinoma cases were included, with 947 in the multiple-tumor group and 18,542 in the single-tumor group. The multiple-tumor group had lower cancer-specific survival (*P* = 0.03, HR = 1.431). Cox regression identified risk factors for the multiple-tumor group including number of tumors, gender, combined summary stage, T stage, N stage, tumor size, and type of surgery. The predicted probabilities showed acceptable agreement with the actual observations at 3-, 5-, and 8-years area under the curve values in both the training and validation cohorts (0.831 vs. 0.605; 0.775 vs. 0.672; and 0.797 vs. 0.699, respectively). Compared with single renal cell carcinoma, multiple renal cell carcinoma is associated with decreased cancer-specific survival. Additionally, we identified several prognostic factors including the number of tumors, T stage, tumor size, and type of surgery. These findings offer valuable insights for selecting appropriate treatment strategies for patients diagnosed with multiple renal cell carcinomas.

**Supplementary Information:**

The online version contains supplementary material available at 10.1007/s10238-024-01433-w.

## Introduction

Renal cell carcinoma (RCC) represents one of the most prevalent renal malignancies globally, with its incidence steadily rising over time [1]. Recognized as the seventh most commonly diagnosed cancer type worldwide by the World Health Organization (WHO), RCC poses a significant health burden [2]. Primary multiple RCC refers to the development of multiple independent tumors within a single patient’s kidneys, either concurrently or at different intervals, without metastasis to other organs. Previous reports indicate that bilateral RCC accounts for approximately 4.3–25% of all cases [3–5], while the detection rate of multiple lesions in unilateral RCC is relatively lower [6].

Existing literature on the pathological homogeneity, treatment strategies, and prognostic factors of multiple RCCs has been predominantly limited to small institutional cohorts, with the largest cohorts to date involving 264 patients [7–9]. It is commonly believed that bilateral multiple renal tumors demonstrate a greater degree of pathological homogeneity [10]. However, the optimal surgical approach remains a topic of ongoing debate and it remains uncertain whether tumor multiplicity influences cancer-specific survival (CSS) or overall survival (OS). While many studies suggest no discernible disparity in survival outcomes between multiple tumors and single tumors, an increased incidence of local recurrence or contralateral recurrence may be observed [6, 7, 9, 11]. Notably, research on prognostic risk factors for patients with multiple RCCs is limited [12].

In this study, we retrieved a large dataset of 19,489 patients from the Surveillance, Epidemiology, and End Results (SEER) database to conduct a comparative analysis of clinical characteristics and survival outcomes between patients with multiple RCCs and those with solitary RCCs. Additionally, we developed and validated a Cox regression model to identify potential prognostic factors in patients diagnosed with multiple RCCs. Our investigation also included evaluating the impact of factors such as the number and laterality of tumors and pathological homogeneity on the prognosis of multiple tumors.

## Methods

### Ethics statement

This retrospective analysis utilized the publicly accessible SEER database, obviating the requirement for informed consent from patients. All procedures adhered to the principles outlined in the Declaration of Helsinki and its subsequent revisions.

### Data sources

Patient data were sourced from the SEER database (SEER*Stat version 8.4.0) of the National Cancer Institute (NCI), globally acknowledged as one of the most comprehensive tumor databases. Covering cancer incidence and survival data for approximately 48% of the American cancer registry population, the SEER database served as a vital resource for this study [13].

### Patient selection

The inclusion criteria were as follows: (1) patients diagnosed with RCC; (2) the absence of distant metastasis (M stage is M0); (3) diagnosis established between the years 2004 and 2015. The exclusion criteria were: (1) diagnosis age < 20 years old; (2) unavailability of baseline data, clinical, and surgical information; (3) lack of confirmation by pathology; (4) inadequate follow-up duration; (5) patients confirmed through autopsy or death certificate (Surgery Codes A000/A990); (6) tumor size not recorded (CS tumor size is coded as 999). A flowchart of patient selection is shown in Online Resource 1 (The flowchart of patient selection). The study included data for a total of 19,489 RCC tumors. Among these cases, 947 were attributed to patients with multiple tumors, while the remaining 18,542 represented single tumors. The information pertaining to multiple tumors was randomly partitioned into a training cohort (n = 662, 70%) and a validation cohort (n = 285, 30%).

### Inclusion variables

The study encompassed the following variables: population characteristics, including age, gender, and race; clinical information such as pathological type, tumor stage, tumor size, type of surgery, number of tumors, laterality (unilateral or bilateral), pathological heterogeneity or homogeneity; and survival-related data, including survival status and survival time. The TNM staging for tumors was based on the 7th edition of the AJCC (American Joint Committee on Cancer) TNM staging system. The types of surgeries included A100 Local tumor destruction, NOS; A200 Local tumor excision, NOS; A300 Partial or subtotal nephrectomy (kidney or renal pelvis); A400 Complete/total/simple nephrectomy—for kidney parenchyma; A500 Radical nephrectomy; A700 Any nephrectomy (simple, subtotal, complete, partial, total, radical) in continuity with the resection of other organ(s) (colon, bladder); A800 Nephrectomy, NOS; and A900 Surgery, NOS. The follow-up period extended until November 2019. Online Resource 2 (Variable assignment) presents a comprehensive overview of the variable assignments.

### Statistical methods

SPSS 26.0 and R software (version 4.2.3) were used for data processing Survival curves were generated using GraphPad Prism 8. Descriptive statistics for basic characteristics are presented as numbers and percentages (n, %). Survival outcomes, considering both CSS and OS, were evaluated. The t test was used for continuous variables and the chi-square test was used for categorical variables, with Fisher’s exact test utilized in situations with low counts. Kaplan–Meier curves were generated and the log-rank test was used for survival analyses. Cox proportional hazards models were constructed to identify potential prognostic factors. Time-dependent receiver operating characteristic (ROC) curves were generated by selecting time points of 3-, 5-, and 8-years to calculate the area under the curve (AUC), which exhibits a positive correlation with predictive accuracy. For all tests, two-sided *P* values < 0.05 were considered to indicate statistical significance.

## Results

### Baseline patient characteristics

The patients’ characteristics are shown in Table 1. Compared to the single-tumor group, the multiple-tumor group exhibited a greater proportion of males (74.87% vs. 62.15%, *P* < 0.001), a lower proportion of Caucasians (70.01% vs. 81.32%, *P* < 0.001), a greater prevalence of pRCC (29.36% vs. 11.99%, *P* < 0.001), an increased occurrence of localized tumors in combined summary staging (88.91% vs. 81.13%, *P* < 0.001), and T and N staging that leaned toward early-stage RCC (*P* < 0.001, *P* < 0.05). The type of surgery tended to favor partial or subtotal nephrectomy (52.59% vs. 30.96%, *P* < 0.001).

**Table 1:** Patient characteristics before and after PSM

Variables	Before PSM				After PSM			
	Total (n = 19,489)	Multiple-tumor group	Single-tumor group	*P* value	Total (n = 1892)	Multiple-tumor group	Single-tumor group	*P* value^a^
		(n = 947)	(n = 18,542)			(n = 946)	(n = 946)	
Age, n (%)				0.787				0.073
< 60 years old	9612 (49.32)	463 (48.89)	9149 (49.34)		963 (50.9)	462 (48.84)	501 (52.96)	
≥ 60 years old	9877 (50.68)	484 (51.11)	9393 (50.66)		929 (49.1)	484 (51.16)	445 (47.04)	
Gender, n (%)				< 0.001				0.873
Male	12,232 (62.76)	709 (74.87)	11,523 (62.15)		1419 (75)	708 (74.84)	711 (75.16)	
Female	7257 (37.24)	238 (25.13)	7019 (37.85)		473 (25)	238 (25.16)	235 (24.84)	
Race, n (%)				< 0.001				0.65
White	15,741 (80.77)	663 (70.01)	15,078 (81.32)		1308 (69.13)	663 (70.08)	645 (68.18)	
Black	1789 (9.18)	211 (22.28)	1578 (8.51)		436 (23.04)	210 (22.20)	226 (23.89)	
Other	1959 (10.05)	73 (7.71)	1886 (10.17)		148 (7.82)	73 (7.72)	75 (7.93)	
Pathological Type, n (%)				< 0.001				0.736
ccRCC	12,556 (64.43)	510 (53.85)	12,046 (64.97)		1022 (54.02)	509 (53.81)	513 (54.23)	
pRCC	2502 (12.84)	278 (29.36)	2224 (11.99)		542 (28.65)	278 (29.39)	264 (27.91)	
Other	3055 (15.68)	118 (12.46)	2937 (15.84)		237 (12.53)	118 (12.47)	119 (12.58)	
chRCC	1376 (7.06)	41 (4.33)	1335 (7.20)		91 (4.81)	41 (4.33)	50 (5.29)	
Combined summary stage, n (%)				< 0.001				0.563
Localized	15,886 (81.51)	842 (88.91)	15,044 (81.13)		1676 (88.58)	842 (89.01)	834 (88.16)	
Regional	3603 (18.49)	105 (11.09)	3498 (18.87)		216 (11.42)	104 (10.99)	112 (11.84)	
T stage, n (%)				< 0.001				0.514
T1	13,770 (70.66)	776 (81.94)	12,994 (70.08)		1530 (80.87)	776 (82.03)	754 (79.70)	
T2	2227 (11.43)	70 (7.39)	2157 (11.63)		155 (8.19)	70 (7.40)	85 (8.99)	
T3	3430 (17.6)	96 (10.14)	3334 (17.98)		197 (10.41)	96 (10.15)	101 (10.68)	
T4	62 (0.32)	5 (0.53)	57 (0.31)		10 (0.53)	4 (0.42)	6 (0.63)	
N stage, n (%)				0.021				0.465
N0	19,147 (98.25)	940 (99.26)	18,207 (98.19)		1875 (99.1)	939 (99.26)	936 (98.94)	
N1	216 (1.11)	7 (0.74)	209 (1.13)		17 (0.9)	7 (0.74)	10 (1.06)	
N2	126 (0.65)	0 (0.00)	126 (0.68)					
Tumor size, n (%)				< 0.001				0.315
≤ 70mm	15,430 (79.17)	843 (89.02)	14,587 (78.67)		220 (11.63)	103 (10.89)	117 (12.37)	
> 70mm	4059 (20.83)	104 (10.98)	3955 (21.33)					
Type of surgery, n (%)				< 0.001				
A100/A200	1084 (5.56)	90 (9.50)	994 (5.36)					
A300	6238 (32.01)	498 (52.59)	5740 (30.96)					
A400	1436 (7.37)	62 (6.55)	1374 (7.41)					
A500	10,467 (53.71)	289 (30.52)	10,178 (54.89)					
A700	112 (0.57)	1 (0.11)	111 (0.60)					
A800/A900	152 (0.78)	7 (0.74)	145 (0.78)					

A100 Local tumor destruction, NOS; A200 Local tumor excision, NOS; A300 Partial or subtotal nephrectomy (kidney or renal pelvis); A400 Complete/total/simple nephrectomy—for kidney parenchyma; A500 Radical nephrectomy; A700 Any nephrectomy (simple, subtotal, complete, partial, total, radical) in continuity with the resection of other organ(s) (colon, bladder); A800 Nephrectomy, NOS; A900 Surgery, NOS

^a^ *P* value based on the χ^2^ test; ccRCC, clear cell renal cell carcinoma; pRCC, papillary renal cell carcinoma; chRCC, chromophobe cell renal carcinoma

### Survival curves

To mitigate potential confounding factors impacting survival analysis, propensity score matching (PSM) was applied, incorporating age, gender, race, pathological type, combined summary stage, T stage, N stage, and tumor size as covariates. Prioritizing nearest matches yielded a total of 946 pairs of tumor information for inclusion in the survival analysis. Table 1 presents the patient characteristics before and after PSM, with no significant difference between the matched groups.

**Fig. 1:**
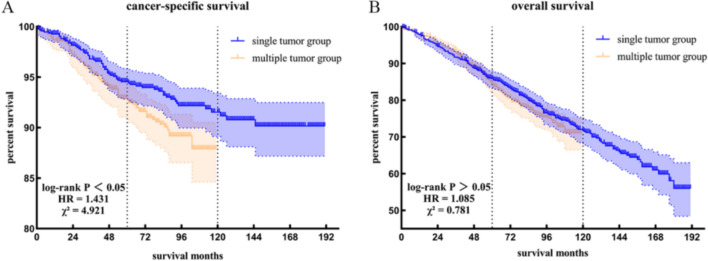
Comparison of survival outcomes using the Kaplan–Meier method. **A** Cancer-specific survival. **B** Overall survival. The blue line represents the single-tumor group. The orange line represents the multiple-tumor group (color figure online)

The Kaplan–Meier method was used to generate survival curves for patients with multiple tumors and those with single tumors (Fig. 1), followed by a log-rank test. The results revealed a statistically significant difference in CSS between the two groups (*P* = 0.03, HR = 1.431, χ^2^ = 4.921), while no statistically significant difference was observed in OS (*P* = 0.38, HR = 1.085, χ^2^ = 0.781). As of the follow-up time, the CSS for the multiple-tumor group was 87.90%, and the OS was 71.10%, whereas for the single-tumor group, the CSS was 90.16%, and the OS was 56.01%.

### Analyses of risk factors for multiple-tumor group

Univariate and multivariate Cox regressions were performed with the training cohort. The number of tumors was treated as a continuous variable, whereas other variables were considered categorical. The results indicated that the number of tumors, gender, combined summary stage, T stage, N stage, tumor size, and type of surgery significantly influenced CSS. Conversely, the pathological type, presence of pathological heterogeneity or homogeneity, and laterality did not exhibit a statistically significant impact (Table 2).

**Table 2:** Univariate and multivariate Cox regressions for analyzing the risk factors for CSS in the multiple-tumor group

Variables	Univariate		Multivariate	
	*P* value	HR (95%CI)	*P* value	HR (95%CI)
Number of tumors				
	0.028	1.83 (1.07–3.14)	0.013	2.09 (1.17–3.76)
Age				
< 60 years old		Ref		
≥ 60 years old	0.711	0.91 (0.55–1.51)		
Gender				
Male		Ref		Ref
Female	0.017	0.38 (0.17–0.85)	0.036	0.43 (0.19–0.94)
Race				
White		Ref		
Black	0.724	0.89 (0.48–1.67)		
Other	0.808	1.11 (0.47–2.62)		
Pathological type				
ccRCC		Ref		
pRCC	0.387	0.76 (0.41–1.41)		
chRCC	0.299	0.35 (0.05–2.55)		
Other	0.58	1.21 (0.61–2.40)		
Combined summary stage				
Localized		Ref		Ref
Regional	< 0.001	5.94 (3.52–10.05)	0.551	0.47 (0.04–5.52)
T stage				
T1		Ref		Ref
T2	0.02	2.52 (1.16–5.48)	0.291	0.53 (0.16–1.73)
T3	< 0.001	6.59 (3.76–11.57)	0.107	7.51 (0.65–87.19)
T4	< 0.001	53.36 (6.62–430.27)	0.027	40.25 (1.53–1055.72)
N stage				
N0		Ref		Ref
N1	< 0.001	6.80 (2.46–18.79)	0.014	4.88 (1.38–17.31)
Tumor Size				
≤ 70mm		Ref		Ref
> 70mm	< 0.001	3.88 (2.25–6.69)	0.001	4.09 (1.74–9.64)
Type of surgery				
A100/A200		Ref		Ref
A300	< 0.001	0.25 (0.11–0.57)	< 0.001	0.19 (0.08–0.43)
A400	0.736	0.84 (0.31–2.31)	0.117	0.42 (0.14–1.25)
A500	0.842	1.08 (0.53–2.20)	0.065	0.45 (0.19–1.05)
A700	0.997	0.00 (0.00–Inf)	0.997	0.00 (0.00–Inf)
A800/A900	0.996	0.00 (0.00–Inf)	0.996	0.00 (0.00–Inf)
Laterality				
Bilateral		Ref		
Unilateral	0.761	0.90 (0.46–1.77)		
Heterogeneity or homogeneity				
Heterogeneity		Ref		
Homogeneity	0.471	0.83 (0.50–1.38)		

A100 Local tumor destruction, NOS; A200 Local tumor excision, NOS; A300 Partial or subtotal nephrectomy (kidney or renal pelvis); A400 Complete/total/simple nephrectomy—for kidney parenchyma; A500 Radical nephrectomy; A700 Any nephrectomy (simple, subtotal, complete, partial, total, radical) in continuity with the resection of other organ(s) (colon, bladder); A800 Nephrectomy, NOS; A900 Surgery, NOS

ccRCC, clear cell renal cell carcinoma; pRCC, papillary renal cell carcinoma; chRCC, chromophobe cell renal carcinoma

### Model visualization and performance evaluation

**Fig. 2:**
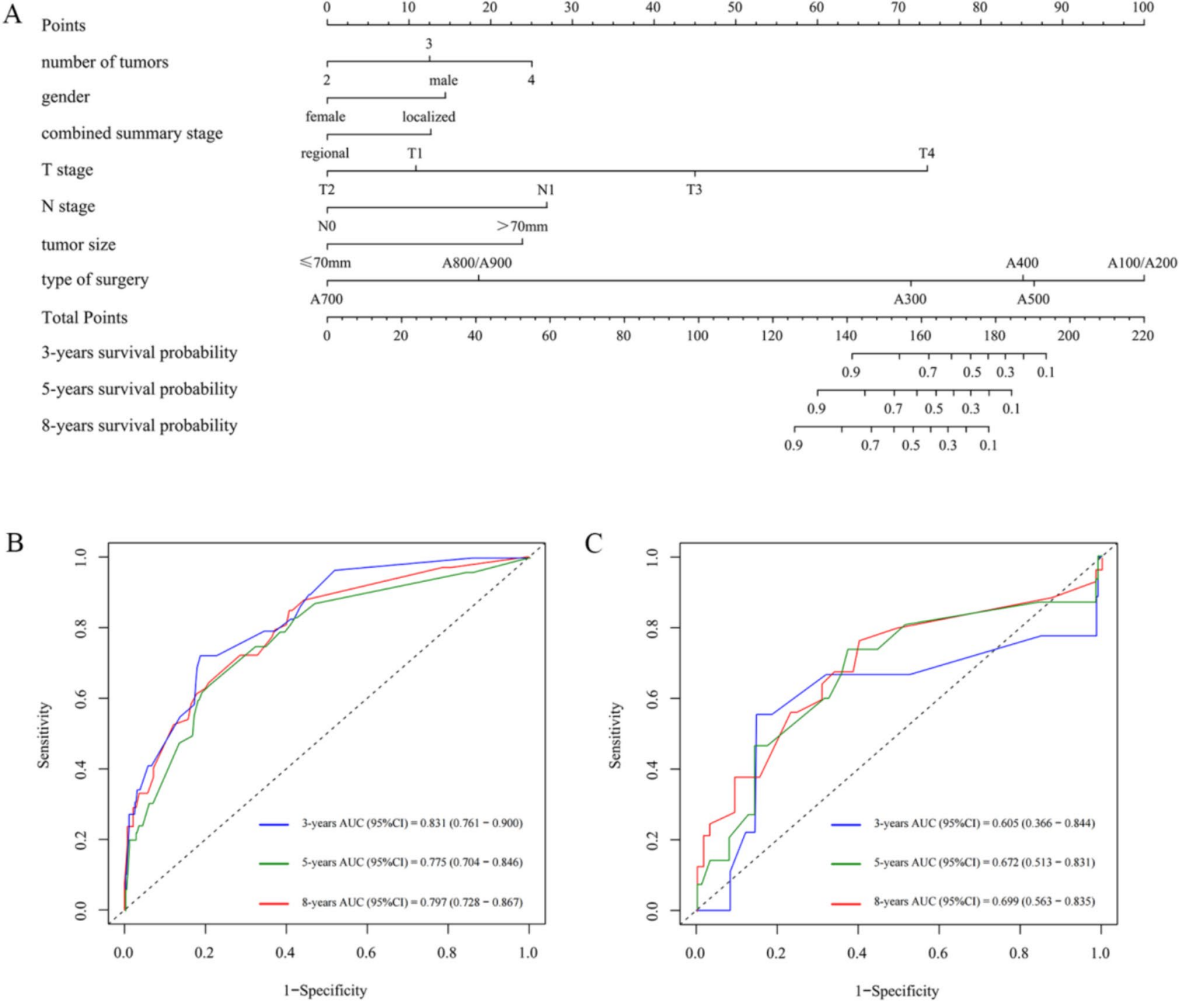
Model visualization and evaluation. **A** Nomogram for 3-, 5-, and 8-year CSS in patients with multiple RCCs. **B** The ROC curves for 3-, 5-, and 8-years survival in the training cohort. **C** The ROC curves for 3-, 5-, and 8-years survival in the validation cohort

The multivariable Cox regression model showed that specific variables were significantly correlated with the CSS of patients in the multiple-tumor group. Nomograms were constructed for CSS, incorporating these significant predictors, including the number of tumors, gender, combined summary stage, T stage, N stage, tumor size, and type of surgery, to visualize the model (Fig. 2A).

The efficiency of the model was further verified in the validation cohort. Online Resource 3 (Comparison of clinical characteristics between the training and validation cohorts, no statistically significant differences were observed) illustrates patient characteristics in the training and validation cohorts, revealing no statistically significant differences. The ROC curves and AUC values were used to evaluate the discriminative ability of the model in the training and validation cohorts. The 3-, 5-, and 8-years AUC values in the training and validation cohort were 0.831 vs. 0.605, 0.775 vs. 0.672, and 0.797 vs. 0.699, respectively (Fig. 2B, C), exhibiting acceptable agreement between the model-predicted and actual observed probabilities.

### Further investigation of the characteristics of multiple tumors

For the management of multiple tumors, crucial factors influencing treatment decisions include the number and laterality (unilateral or bilateral) of tumors, as well as the homogeneity observed in tumor pathology. Therefore, a thorough investigation into these aspects is imperative to enhance our understanding and guide optimal treatment strategies.

Considering the aforementioned characteristics, patients presenting with multiple tumors can be classified into eight distinct subgroups. Kaplan–Meier curves show noteworthy variations in CSS and OS among the subgroups, even in instances where no observed death events were noted in certain subgroups due to the limited sample size. Notably, patients with bilateral three-pathological heterogeneous tumors are associated with an elevated risk of survival (Online Resource 4 Comparison of survival outcomes among subgroups using the Kaplan–Meier method).

**Fig. 3:**
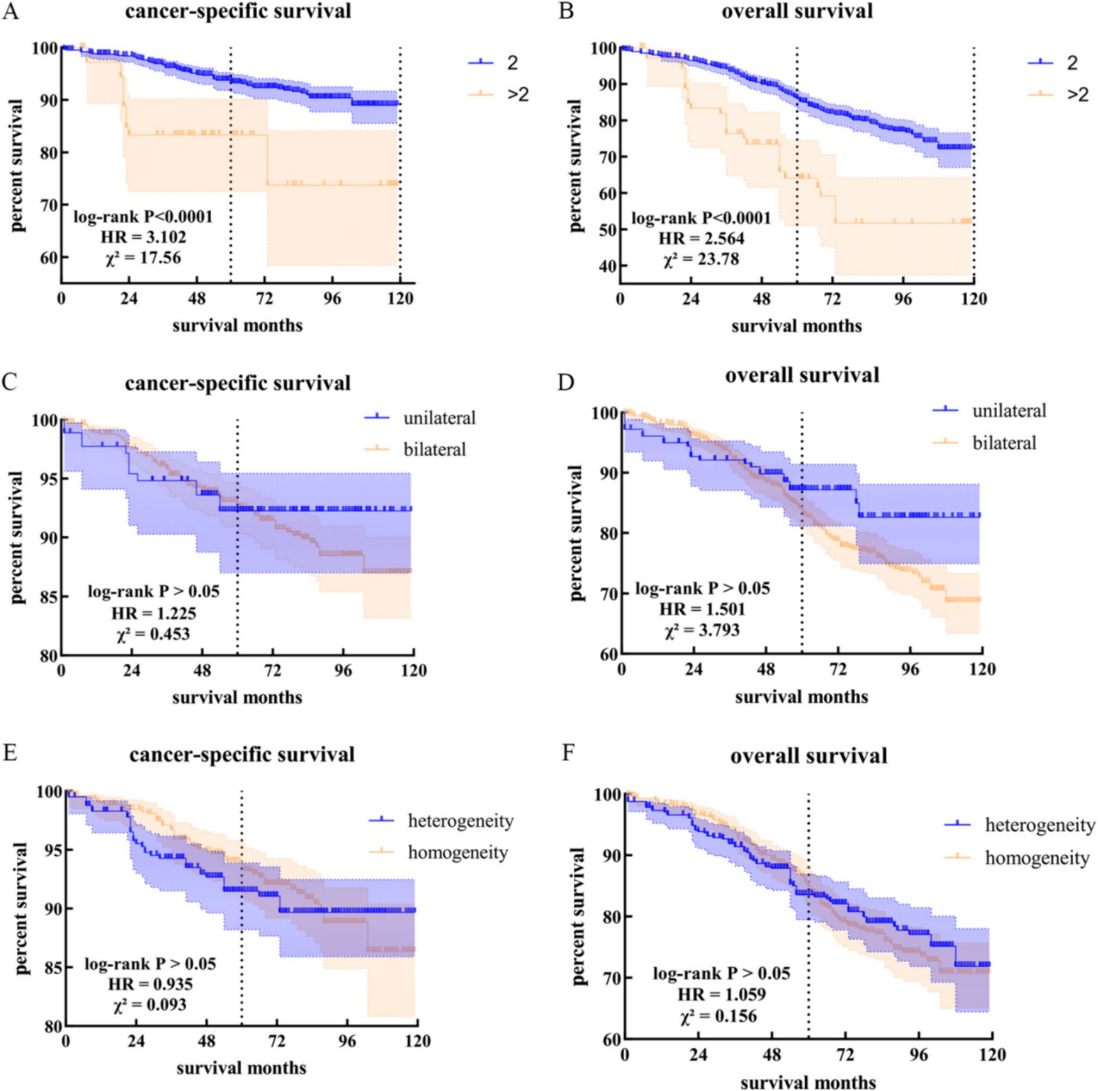
Exploring impact factors: CSS and OS analysis. **A** Cancer-specific survival between patients with two tumors and with more than two tumors. **B** Overall survival between patients with two tumors and with more than two tumors. **C** Cancer-specific survival between patients with unilateral tumors and with bilateral tumors. **D** Overall survival between patients with unilateral tumors and with bilateral tumors. **E** Cancer-specific survival between patients with pathological heterogeneous tumors and with pathological homogeneous tumors. **F** Overall survival between patients with pathological heterogeneous tumors and with pathological homogeneous tumors

In addition, we investigated the impact of each factor on both CSS and OS. The number of tumors significantly influenced CSS and OS outcomes. However, factors such as laterality and pathological heterogeneity or homogeneity did not have any statistically significant effect (Fig. 3).

By analyzing pathological homogeneity within the unilateral and bilateral subgroups, we found that among the 91 cases in the unilateral subgroup, 8 (8.79%) exhibited the same pathological type, while 83 (91.21%) had different pathological types. In contrast, among 369 cases in the bilateral subgroup, 256 cases (69.38%) had the same pathological type, while 113 cases (30.62%) had different pathological types. Pathological heterogeneity was higher in the unilateral subgroup, whereas the bilateral subgroup tended to exhibit consistent pathological types (*P* < 0.001).

## Discussion

Despite the relatively low incidence rate, which has been reported to range from 4.3% to 25% in previous studies and was found to be 4.86% in this study [3], discernible disparities exist in the clinical characteristics between patients who develop multiple RCCs and those with a single RCC [6, 7, 11]. Moreover, the ongoing debate regarding the impact of tumor multifocality on prognosis persists. Previous studies have suggested that there is no significant disparity in CSS between individuals diagnosed with either a single or multiple tumors; however, cases involving multifocal lesions tend to exhibit higher rates of local recurrence or contralateral recurrence [6, 11, 14–16].

In our extensive investigation with a substantial sample size, comparable overall survival rates were observed between the two groups. However, it is crucial to acknowledge that patients with multiple RCCs exhibit diminished CSS. These disparities may stem from variations in sample size or surgical intervention preferences during treatment planning for these specific patient cohorts, emphasizing the need for a more holistic approach in treatment modalities and patient management.

In contrast to prior studies, our findings indicate that multiple RCCs are associated with lower CSS. Cox regression analysis further revealed that the surgical approach was the most significant factor, with gender, tumor stage, and tumor size also significantly impacting CSS. Among the unique characteristics of multiple tumors, only the number of tumors affected the survival prognosis. Due to the limited number of patients with more than two tumors, we were unable to confirm whether survival risk was positively correlated with the number of tumors. In our dataset, patients with three bilateral heterogeneous tumors had a higher risk of survival.

In the domain of clinical diagnosis and treatment, the existence of multiple tumors undeniably mandates nephron-sparing surgery, thereby presenting a formidable challenge. Multiple renal tumor surgeries are associated with increased complication rates, significantly prolonged hot ischemia times, significantly reduced postoperative eGFR (estimated Glomerular Filtration Rate) level, and higher surgical difficulty and risk [7, 17–19]. This study unequivocally underscores the paramount significance of the type of surgery. For the treatment of multiple RCCs, individualized considerations are necessary to ultimately optimize patient outcomes. For patients with early tumor staging, small tumors, and a limited number of tumors, partial nephrectomy represents a viable option. In the case of patients with advanced tumor staging, large tumors, or more than two tumors, the implementation of preoperative neoadjuvant therapy or postoperative adjuvant therapy can effectively mitigate the tumor burden and minimize the risk of recurrence. New auxiliary technologies such as IQQA (three-dimensional intelligent qualitative and quantitative analysis system), 3D printing, and near-infrared fluorescence imaging [20–22], along with improved surgical procedures such as early unclamping, segmental clamping, and superselective clamping [23, 24], are beneficial for patients to achieve the “trifecta” outcomes: negative cancer margin, minimal renal functional decrease and no urological complications [25]. For surgeries of high complexity, open surgery is also a viable option [9].

Multiple bilateral multiple tumors exhibited higher pathological homogeneity, while multiple unilateral tumors tended to have different pathological types, consistent with previous studies [8, 10]. This suggests that for patients with multiple unilateral renal tumors, a single area of pathological examination may not effectively guide treatment. For patients with multiple bilateral renal tumors, given their high pathological consistency, after histological confirmation of one lesion, more conservative treatment methods such as needle biopsy and local destruction can be selected for the other lesion [26].

The potential oversight of undetected satellite cancerous foci after resection of the primary lesion poses a risk to oncological outcomes [3]. The elevated rate of contralateral recurrence also presents substantial management challenges [6, 15, 16]. Postoperative surveillance should involve meticulous scrutiny of the ipsilateral kidney and vigilant monitoring for emerging masses in the contralateral renal unit. This comprehensive approach ensures timely detection of potential complications, optimizing patient outcomes.

This study is limited by its reliance on data from the SEER database, representing region-specific registries and may not fully capture the global population with multiple renal cancers. The absence of information on chemotherapy limits the discussion of comprehensive treatment options. The relatively small number of patients with multiple RCCs and the uneven distribution of T stage and N stage may not fully reflect certain differences. Larger-scale studies are necessary to validate and enhance the reliability of the conclusions. Moreover, the SEER database does not provide information on the global volume of tumor burden, which could have been a valuable metric for comparing multiple versus single RCCs. This is a significant limitation that we acknowledge in our study. Additionally, the study does not differentiate between synchronous and metachronous groups based on the time gap of tumor diagnosis, requiring further analysis for a deeper understanding.

In conclusion**,** this study revealed notable disparities in clinical characteristics between patients with multiple RCCs and those with a single RCC, with individuals with multiple RCCs demonstrating diminished CSS. Prognostic factors associated with CSS in multiple RCCs were identified. The distinctive attributes of multiple tumors were explored, providing valuable insights for comprehending these features and optimizing management. Future research should prioritize incorporating more comprehensive data on multiple RCCs, delving deeper into their pathological characteristics, and exploring individualized treatment strategies.

## Supplementary Information

Below is the link to the electronic supplementary material.

Supplementary file1 (PDF 186 KB)

Supplementary file3 (XLSX 11 KB)

Supplementary file4 (XLSX 14 KB)

Supplementary file2 (PDF 184 KB)

## Abbreviations

RCC: Renal cell carcinoma
SEER: Surveillance, epidemiology, and end results
HR: Hazard rate
AUC: Area under the curve
CSS: Cancer-specific Survival
WHO: World Health Organization
OS: Overall survival
NCI: National cancer institute
AJCC: American joint committee on cancer
NOS: Not otherwise specified
ROC: Receiver operating characteristic
PSM: Propensity score matching
IQQA: Three-dimensional Intelligent qualitative and quantitative analysis system
eGFR: Estimated glomerular filtration rate
ccRCC: Clear cell renal cell carcinoma
pRCC: Papillary renal cell carcinoma
chRCC: Chromophobe cell renal carcinoma

## Declarations

### Data availability

The data used in this analysis can be obtained from the SEER database with the use of reasonable procedures.
