# Supplementary material for: Impact of tumor multiplicity on the prognosis of patients with primary renal cell carcinoma: a SEER database analysis
Source: Clin Exp Med. 2024 Aug 17;24(1):194. doi: 10.1007/s10238-024-01433-w (PMC11330414; doi:10.1007/s10238-024-01433-w)
Supplement: Supplementary file 1 — Supplementary file1 (PDF 186 KB) [file 10238_2024_1433_MOESM1_ESM.pdf]

Impact of Tumor Multiplicity on the Prognosis of Patients with Primary Renal Cell Carcinoma: A SEER Database Analysis

Tianyue Yang†[1], Hongfeng Zheng†[1], Shaojun Chen†[1], Min Gong†[2], Yifan Liu[1], Wang Zhou[1], Jianqing Ye*[1], Xiuwu Pan*[1], Xingang Cui*[1]

[1]Department of Urology, Xinhua Hospital, School of Medicine, Shanghai Jiao Tong University, 1665 Kongjiang Road, Shanghai, China, 200092

[2]Department of Urology, Seventh People's Hospital of Shanghai University of Traditional Chinese Medicine, Shanghai, China, 200137

†These authors contributed equally to this paper.

**Corresponding Authors:*

*Xin-gang Cui, Mailing address:1665 Kongjiang Road, Yangpu District, Shanghai, China,200092, Xinhua Hospital, School of Medicine, Shanghai Jiao Tong University; Telephone number: 021-25078090; Fax number: 021-65795173; E-mail address: cuixingang@xinhuamed.com.cn; ORCID: 0000-0002-0920-9439

*Xiuwu Pan, Mailing address:1665 Kongjiang Road, Yangpu District, Shanghai, China,200092, Xinhua Hospital, School of Medicine, Shanghai Jiao Tong University; Telephone number: 021-25078090; Fax number: 021-65795173; Email: panxiuwu@126.com;

*Jianqing Ye, Mailing address:1665 Kongjiang Road, Yangpu District, Shanghai, China,200092, Xinhua Hospital, School of Medicine, Shanghai Jiao Tong University; Telephone number: 021-25078090; Fax number: 021-65795173; Email: ye910@126.com

Supplemental Figure 1

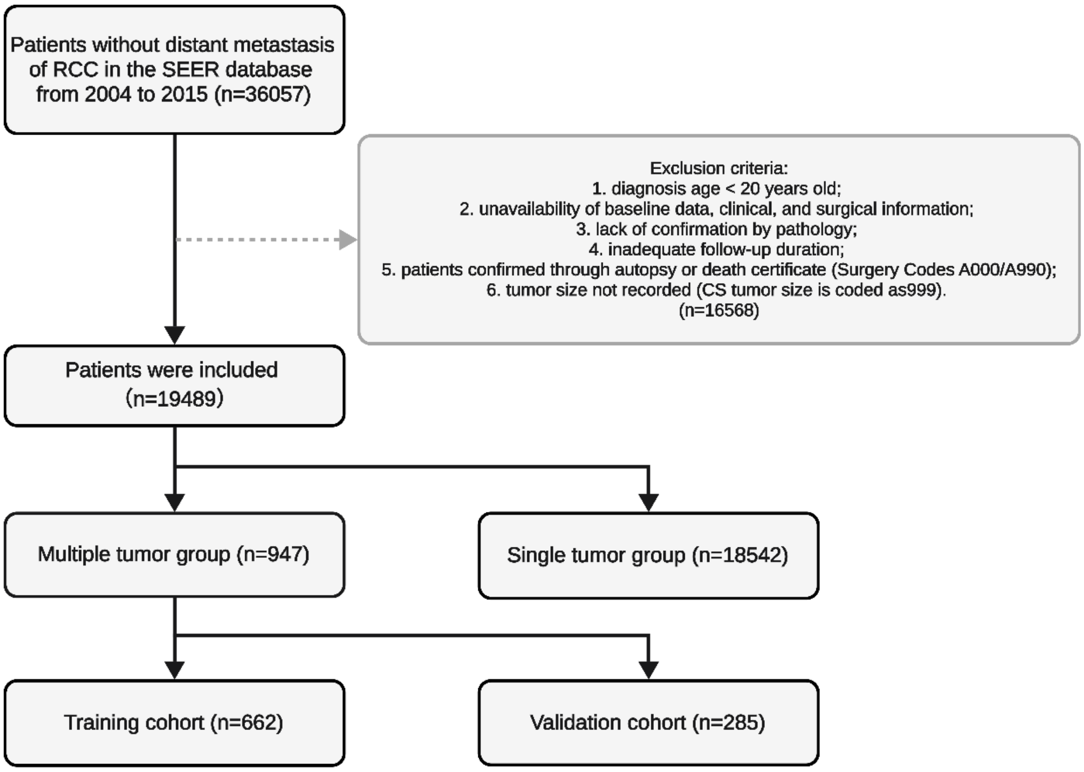

The flowchart of patient selection.
