# Supplementary material for: Impact of tumor multiplicity on the prognosis of patients with primary renal cell carcinoma: a SEER database analysis
Source: Clin Exp Med. 2024 Aug 17;24(1):194. doi: 10.1007/s10238-024-01433-w (PMC11330414; doi:10.1007/s10238-024-01433-w)
Supplement: Supplementary file 4 — Supplementary file2 (PDF 184 KB) [file 10238_2024_1433_MOESM4_ESM.pdf]

Impact of Tumor Multiplicity on the Prognosis of Patients with Primary Renal Cell Carcinoma: A SEER Database Analysis

Tianyue Yang†1, Hongfeng Zheng†1, Shaojun Chen†1, Min Gong†2, Yifan Liu[1], Wang Zhou[1], Jianqing Ye*1, Xiuwu Pan*1, Xingang Cui*1

[1]Department of Urology, Xinhua Hospital, School of Medicine, Shanghai Jiao Tong University, 1665 Kongjiang Road, Shanghai, China, 200092

[2]Department of Urology, Seventh People's Hospital of Shanghai University of Traditional Chinese Medicine, Shanghai, China, 200137

†These authors contributed equally to this paper.

**Corresponding Authors:*

*Xin-gang Cui, Mailing address:1665 Kongjiang Road, Yangpu District, Shanghai, China,200092, Xinhua Hospital, School of Medicine, Shanghai Jiao Tong University; Telephone number: 021-25078090; Fax number: 021-65795173; E-mail address: cuixingang@xinhuamed.com.cn; ORCID: 0000-0002-0920-9439

*Xiuwu Pan, Mailing address:1665 Kongjiang Road, Yangpu District, Shanghai, China,200092, Xinhua Hospital, School of Medicine, Shanghai Jiao Tong University; Telephone number: 021-25078090; Fax number: 021-65795173; Email: panxiuwu@126.com;

*Jianqing Ye, Mailing address:1665 Kongjiang Road, Yangpu District, Shanghai, China,200092, Xinhua Hospital, School of Medicine, Shanghai Jiao Tong University; Telephone number: 021-25078090; Fax number: 021-65795173; Email: ye910@126.com

Supplemental Figure 2 Comparison of survival outcomes among subgroups using the Kaplan-Meier method

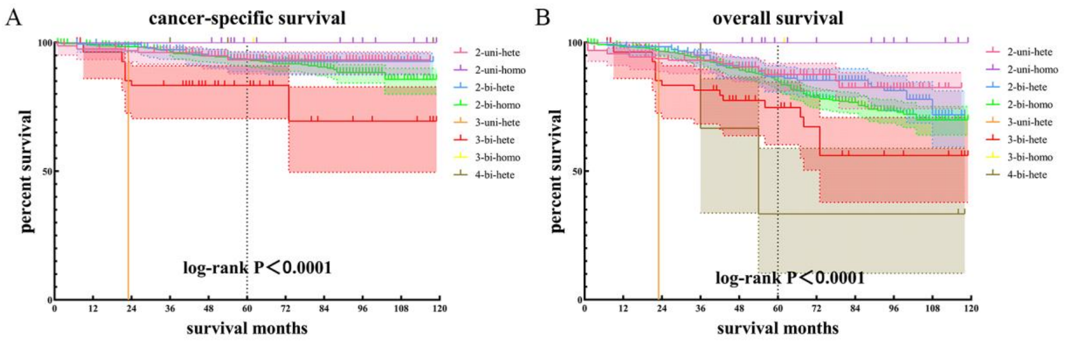

A, Cancer-specific survival. B, Overall survival.

The pink line represents patients with two-unilateral-heterogeneity tumors (n=82, 17.83%).

The purple line represents patients with two-unilateral-homogeneity tumors (n=8, 1.74%).

The blue line represents patients with two-bilateral-heterogeneity tumors (n=91,19.78%).

The green line represents patients with two-bilateral-homogeneity tumors (n=255, 55.43%).

The orange line represents patients with three-unilateral-heterogeneity tumors (n=1,0.22%).

The red line represents patients with three-bilateral-heterogeneity tumors (n=19, 4.13%).

The yellow line represents patients with three-bilateral-homogeneity tumors (n=1, 0.22%).

The brown line represents patients with four-bilateral-heterogeneity tumors (n=3, 0.65%).
